# (*E*)-3-(6-Nitro­benzo[*d*][1,3]dioxol-5-yl)-1-(2,4,6-trimethoxy­phen­yl)prop-2-en-1-one

**DOI:** 10.1107/S1600536809036435

**Published:** 2009-09-26

**Authors:** Hossein Loghmani-Khouzani, Noorsaadah Abdul Rahman, Ward T. Robinson, Marzieh Yaeghoobi, Reza Kia

**Affiliations:** a Chemistry Department, University of Isfahan, Isfahan, 81746-73441, Iran; b University of Malaya, Department of Chemistry, 50603, Kuala Lumpur, Malaysia; c Department of Chemistry, Science and Research Campus, Islamic Azad University, Poonak, Tehran, Iran

## Abstract

In the mol­ecule of the title compound, C_19_H_17_NO_8_, the benzodioxole unit is oriented at a dihedral angle of 61.45 (6)° with respect to the meth­oxy-substituted phenyl ring. The nitro group is not co-planar to the benzene ring to which it is attached, making a dihedral angle of 31.86 (17)°. In the crystal structure, inter­molecular C—H⋯O inter­actions link the mol­ecules into chains through *R* ^2^_2_(8) ring motifs. The π⋯π contacts between the benzodioxole rings, [centroid–centroid distances = 3.7610 (9), 3.6613 (9) and 3.7975 (9) Å] may further stabilize the structure.

## Related literature

For general background to synthesis, see: Nielsen & Houlihan (1968 ▶); Ko *et al.* (2003 ▶); Go *et al.* (2005 ▶); Nowakowska (2007 ▶). For related structures, see: Lawrence *et al.* (2006 ▶); Liu *et al.* (2002 ▶). For ring motifs, see: Bernstein *et al.* (1995 ▶).
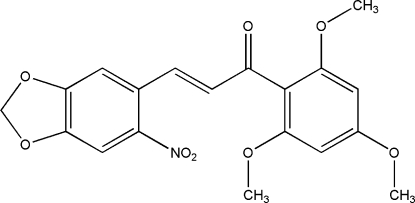


## Experimental

### 

#### Crystal data

C_19_H_17_NO_8_
*M* *_r_* = 387.34
Triclinic, 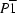

*a* = 7.3044 (1) Å
*b* = 10.1264 (1) Å
*c* = 12.8600 (2) Å
α = 93.112 (1)°
β = 103.959 (1)°
γ = 105.384 (1)°
*V* = 882.91 (2) Å^3^
*Z* = 2
Mo *K*α radiation
μ = 0.12 mm^−1^
*T* = 296 K
0.24 × 0.14 × 0.10 mm

#### Data collection

Bruker SMART APEXII CCD area-detector diffractometer
Absorption correction: multi-scan (*SADABS*; Bruker, 2005 ▶) *T*_min_ = 0.973, *T*_max_ = 0.989
4805 measured reflections
3038 independent reflections
2733 reflections with *I* > 2σ(*I*)
*R*_int_ = 0.013

#### Refinement

*R*[*F* ^2^ > 2σ(*F* ^2^)] = 0.032
*wR*(*F* ^2^) = 0.083
*S* = 1.04
3038 reflections
256 parameters
H-atom parameters constrained
Δρ_max_ = 0.21 e Å^−3^
Δρ_min_ = −0.22 e Å^−3^

### 

Data collection: *APEX2* (Bruker, 2005 ▶); cell refinement: *SAINT* (Bruker, 2005 ▶); data reduction: *SAINT*; program(s) used to solve structure: *SHELXTL* (Sheldrick, 2008 ▶); program(s) used to refine structure: *SHELXTL*; molecular graphics: *SHELXTL*; software used to prepare material for publication: *SHELXTL* and *PLATON* (Spek, 2009 ▶).

## Supplementary Material

Crystal structure: contains datablocks global, I. DOI: 10.1107/S1600536809036435/hk2765sup1.cif

Structure factors: contains datablocks I. DOI: 10.1107/S1600536809036435/hk2765Isup2.hkl

Additional supplementary materials:  crystallographic information; 3D view; checkCIF report

## Figures and Tables

**Table 1:** Hydrogen-bond geometry (Å, °)

*D*—H⋯*A*	*D*—H	H⋯*A*	*D*⋯*A*	*D*—H⋯*A*
C2—H2*A*⋯O1^i^	0.93	2.55	3.4512 (16)	164
C6—H6*A*⋯O6^ii^	0.93	2.36	3.2429 (16)	158

Symmetry codes: (i) 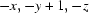; (ii) 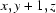.

**Fig. 1.**
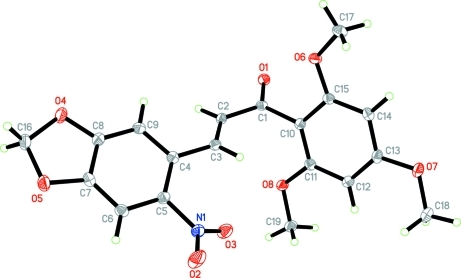
The molecular structure of the title molecule, with the atom-numbering scheme. Displacement ellipsoids are drawn at the 40% probability level.

**Fig. 2.**
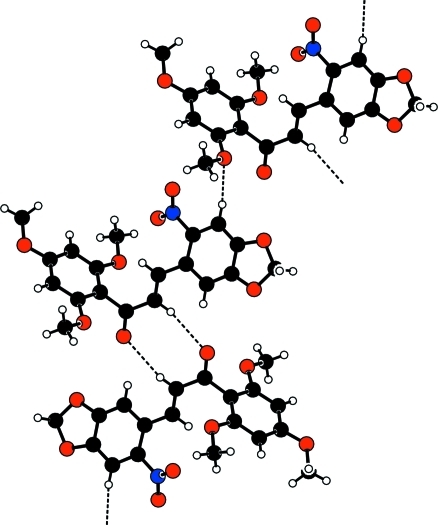
A partial packing diagram. Hydrogen bonds are shown as dashed lines.

## supplementary crystallographic information

### Comment

Aldol condensation reactions are important synthetic reactions and by classical methods they were performed in the presence of strong bases (Nielsen & Houlihan, 1968). Chalcones are open chain flavonides consisting of two aromatic rings linked by an α,β-unsaturated keton moiety. Chalcones have shown a wide variety of anticancer (Lawrence *et al.*,  2006), anti-inflammatory (Ko *et al.*,  2003), antimicrobial (Go *et al.*,  2005) and antifungal (Nowakowska, 2007) activies. Crystal structures of some Chalcones were reported (Lawrence *et al.*, 2006; Liu *et al.*,  2002). They showed the Chalcone molecules are in *s*-trans conformation.

In the molecule of the title compound, (Fig. 1), a new chalcone derivative, the dihedral angle between the benzodioxole ring and the methoxy-substituted phenyl ring is 61.45 (6)°. The nitro-group is tilted with respect to the benzene ring to which it is attached by a dihedral angle of 31.86 (17)°.

In the crystal structure, intermolecular C-H···O interactions link the molecules into chains through *R*^2^_2_(8) ring motifs (Bernstein *et al.*, 1995) (Fig. 2), in which they may be effective in the stabilization of the structure. The π···π contacts between the benzodioxole rings, Cg1—Cg2^i^, Cg2—Cg2^i^ and Cg2—Cg2^ii^ [symmetry codes: (i) -x, 2 - y, -z, (ii) 1 - x, 2 - y, -z, where Cg1 and Cg2 are centroids of the rings (O4/O5/C7/C8/C16) and (C4-C9), respectively] may further stabilize the structure, with centroid-centroid distances of 3.7610 (9), 3.6613 (9) and 3.7975 (9) Å, respectively.

### Experimental

The title compound was obtained according to a literature method (Lawrence *et al.*,  2006), and crystallized in glacial acetic acid. Pale yellow, solid; m.p. 477-479 K; ^1^H NMR (400 MHz; CDCl_3_): δ 7.70 (d, 1H, J = 16 Hz), 7.50 (s, 1H), 7.24 (s, 1H), 6.70 (d, 1H, J = 16 Hz), 6.19 (s, 2H), 6.08 (s, 2H), 3.88 (s, 3H), 3.76 (s, 6H). ^13^C NMR (126 MHz; CDCl_3_): δ 194.3 (C═O), 162.3 (C), 160.0(C), 151.9 (C), 148.0 (C), 146.0 (C), 145.0 (C), 142.9 (C), 140.6 (C), 132.6 (C), 120.0 (CH), 114.3 (C), 107.5 (CH), 105.6 (CH), 103.3 (CH), 90.5 (CH_2_), 55.0 (3CH_3_). IR (KBr, cm^-1^ ): 3015, 1647 (C═O), 1605, 1492, 1511, 1488, 891, 823, 762, 649.

### Refinement

H atoms were positioned geometrically with C-H = 0.93, 0.97 and 0.96 Å for aromatic, methylene and methyl H atoms, respectively, and constrained to ride on their parent atoms, with U_iso_(H) = xU_eq_(C), where x = 1.5 for methyl H and x = 1.2 for all other H atoms.

### Crystal data

**Table:**

C_19_H_17_NO_8_	*Z* = 2
*M**_r_* = 387.34	*F*(000) = 404
Triclinic, *P*1	*D*_x_ = 1.457 Mg m^−^^3^
Hall symbol: -P 1	Melting point: 497 K K
*a* = 7.3044 (1) Å	Mo *K*α radiation, λ = 0.71073 Å
*b* = 10.1264 (1) Å	Cell parameters from 3206 reflections
*c* = 12.8600 (2) Å	θ = 2.5–32.5°
α = 93.112 (1)°	µ = 0.12 mm^−^^1^
β = 103.959 (1)°	*T* = 296 K
γ = 105.384 (1)°	Prism, pale yellow
*V* = 882.91 (2) Å^3^	0.24 × 0.14 × 0.10 mm

### Data collection

**Table:**

Bruker SMART APEXII CCD area-detector diffractometer	3038 independent reflections
Radiation source: fine-focus sealed tube	2733 reflections with *I* > 2σ(*I*)
graphite	*R*_int_ = 0.013
φ and ω scans	θ_max_ = 25.0°, θ_min_ = 2.1°
Absorption correction: multi-scan (*SADABS*; Bruker, 2005)	*h* = −8→7
*T*_min_ = 0.973, *T*_max_ = 0.989	*k* = −11→12
4805 measured reflections	*l* = −15→15

### Refinement

**Table:**

Refinement on *F*^2^	Primary atom site location: structure-invariant direct methods
Least-squares matrix: full	Secondary atom site location: difference Fourier map
*R*[*F*^2^ > 2σ(*F*^2^)] = 0.032	Hydrogen site location: inferred from neighbouring sites
*wR*(*F*^2^) = 0.083	H-atom parameters constrained
*S* = 1.04	*w* = 1/[σ^2^(*F*_o_^2^) + (0.0394*P*)^2^ + 0.3499*P*] where *P* = (*F*_o_^2^ + 2*F*_c_^2^)/3
3038 reflections	(Δ/σ)_max_ < 0.001
256 parameters	Δρ_max_ = 0.21 e Å^−^^3^
0 restraints	Δρ_min_ = −0.22 e Å^−^^3^

### Special details

**Table:**

Experimental. The low-temperature data was collected with the Oxford Cyrosystem Cobra low-temperature attachment.
Geometry. All esds (except the esd in the dihedral angle between two l.s. planes) are estimated using the full covariance matrix. The cell esds are taken into account individually in the estimation of esds in distances, angles and torsion angles; correlations between esds in cell parameters are only used when they are defined by crystal symmetry. An approximate (isotropic) treatment of cell esds is used for estimating esds involving l.s. planes.
Refinement. Refinement of F^2^ against ALL reflections. The weighted R-factor wR and goodness of fit S are based on F^2^, conventional R-factors R are based on F, with F set to zero for negative F^2^. The threshold expression of F^2^ > 2sigma(F^2^) is used only for calculating R-factors(gt) etc. and is not relevant to the choice of reflections for refinement. R-factors based on F^2^ are statistically about twice as large as those based on F, and R- factors based on ALL data will be even larger.

### Fractional atomic coordinates and isotropic or equivalent isotropic displacement parameters (Å^2^)

**Table:**

	*x*	*y*	*z*	*U*_iso_*/*U*_eq_	
O1	0.15752 (13)	0.44029 (9)	0.12868 (7)	0.0209 (2)	
O2	0.54955 (19)	1.20445 (11)	0.27950 (9)	0.0430 (3)	
O3	0.37219 (16)	1.00784 (10)	0.30427 (8)	0.0309 (3)	
O4	0.11057 (18)	1.00352 (10)	−0.19561 (8)	0.0355 (3)	
O5	0.21032 (16)	1.22795 (10)	−0.11005 (8)	0.0298 (3)	
O6	0.58160 (13)	0.48499 (9)	0.17340 (7)	0.0198 (2)	
O7	0.86143 (14)	0.63490 (10)	0.54849 (7)	0.0249 (2)	
O8	0.23749 (13)	0.69600 (10)	0.35796 (7)	0.0210 (2)	
N1	0.42880 (18)	1.09155 (12)	0.24520 (9)	0.0240 (3)	
C1	0.25053 (18)	0.55935 (13)	0.16689 (10)	0.0163 (3)	
C2	0.20715 (19)	0.67463 (13)	0.11028 (10)	0.0180 (3)	
H2A	0.0969	0.6553	0.0515	0.022*	
C3	0.31798 (19)	0.80537 (13)	0.13907 (10)	0.0186 (3)	
H3A	0.4224	0.8247	0.2010	0.022*	
C4	0.28680 (19)	0.92101 (13)	0.07998 (10)	0.0185 (3)	
C5	0.34552 (19)	1.05839 (14)	0.12818 (10)	0.0191 (3)	
C6	0.3278 (2)	1.17065 (14)	0.07231 (11)	0.0210 (3)	
H6A	0.3692	1.2607	0.1069	0.025*	
C7	0.24566 (19)	1.13955 (14)	−0.03640 (11)	0.0207 (3)	
C8	0.1851 (2)	1.00527 (14)	−0.08729 (11)	0.0224 (3)	
C9	0.2033 (2)	0.89551 (14)	−0.03263 (11)	0.0217 (3)	
H9A	0.1616	0.8063	−0.0688	0.026*	
C10	0.41339 (19)	0.58819 (13)	0.26927 (10)	0.0169 (3)	
C11	0.40319 (19)	0.65461 (13)	0.36469 (10)	0.0177 (3)	
C12	0.55162 (19)	0.67427 (13)	0.46034 (10)	0.0193 (3)	
H12A	0.5443	0.7196	0.5234	0.023*	
C13	0.71059 (19)	0.62434 (13)	0.45897 (11)	0.0195 (3)	
C14	0.72776 (19)	0.55933 (13)	0.36521 (11)	0.0194 (3)	
H14A	0.8367	0.5281	0.3656	0.023*	
C15	0.57876 (19)	0.54221 (13)	0.27107 (10)	0.0169 (3)	
C16	0.1199 (2)	1.14409 (14)	−0.21227 (11)	0.0261 (3)	
H16A	0.1968	1.1736	−0.2631	0.031*	
H16B	−0.0115	1.1523	−0.2414	0.031*	
C17	0.7629 (2)	0.46353 (15)	0.16380 (12)	0.0242 (3)	
H17A	0.7548	0.4414	0.0890	0.036*	
H17B	0.7868	0.3887	0.2025	0.036*	
H17C	0.8687	0.5460	0.1934	0.036*	
C18	0.8713 (2)	0.72180 (16)	0.64213 (12)	0.0313 (4)	
H18A	0.9939	0.7326	0.6950	0.047*	
H18B	0.7640	0.6807	0.6718	0.047*	
H18C	0.8627	0.8105	0.6226	0.047*	
C19	0.2283 (2)	0.77177 (15)	0.45311 (11)	0.0245 (3)	
H19A	0.1095	0.7994	0.4380	0.037*	
H19B	0.3401	0.8522	0.4749	0.037*	
H19C	0.2289	0.7144	0.5102	0.037*	

### Atomic displacement parameters (Å^2^)

**Table:**

	*U*^11^	*U*^22^	*U*^33^	*U*^12^	*U*^13^	*U*^23^
O1	0.0207 (5)	0.0174 (5)	0.0213 (5)	0.0041 (4)	0.0013 (4)	−0.0011 (4)
O2	0.0559 (8)	0.0233 (6)	0.0267 (6)	−0.0099 (5)	−0.0077 (5)	−0.0010 (5)
O3	0.0430 (6)	0.0253 (5)	0.0209 (5)	0.0040 (5)	0.0081 (5)	0.0036 (4)
O4	0.0602 (8)	0.0213 (5)	0.0176 (5)	0.0108 (5)	−0.0022 (5)	0.0031 (4)
O5	0.0397 (6)	0.0189 (5)	0.0248 (5)	0.0073 (4)	−0.0019 (4)	0.0049 (4)
O6	0.0191 (5)	0.0208 (5)	0.0201 (5)	0.0070 (4)	0.0056 (4)	−0.0017 (4)
O7	0.0214 (5)	0.0317 (5)	0.0189 (5)	0.0096 (4)	−0.0015 (4)	0.0012 (4)
O8	0.0195 (5)	0.0269 (5)	0.0177 (5)	0.0104 (4)	0.0037 (4)	−0.0013 (4)
N1	0.0283 (7)	0.0188 (6)	0.0209 (6)	0.0050 (5)	0.0013 (5)	0.0001 (5)
C1	0.0148 (6)	0.0180 (7)	0.0170 (6)	0.0045 (5)	0.0063 (5)	−0.0001 (5)
C2	0.0171 (7)	0.0220 (7)	0.0151 (6)	0.0077 (5)	0.0028 (5)	0.0012 (5)
C3	0.0184 (7)	0.0216 (7)	0.0160 (6)	0.0075 (5)	0.0036 (5)	0.0005 (5)
C4	0.0163 (7)	0.0189 (7)	0.0203 (7)	0.0048 (5)	0.0049 (5)	0.0021 (5)
C5	0.0165 (7)	0.0207 (7)	0.0175 (7)	0.0033 (5)	0.0023 (5)	0.0000 (5)
C6	0.0195 (7)	0.0158 (7)	0.0250 (7)	0.0037 (5)	0.0033 (6)	−0.0006 (5)
C7	0.0193 (7)	0.0184 (7)	0.0250 (7)	0.0065 (5)	0.0049 (6)	0.0055 (5)
C8	0.0245 (7)	0.0233 (7)	0.0176 (7)	0.0069 (6)	0.0025 (6)	0.0014 (5)
C9	0.0268 (7)	0.0165 (7)	0.0204 (7)	0.0059 (6)	0.0044 (6)	−0.0006 (5)
C10	0.0183 (7)	0.0137 (6)	0.0169 (6)	0.0026 (5)	0.0036 (5)	0.0027 (5)
C11	0.0170 (7)	0.0157 (6)	0.0201 (7)	0.0042 (5)	0.0047 (5)	0.0029 (5)
C12	0.0209 (7)	0.0187 (7)	0.0171 (6)	0.0042 (5)	0.0050 (5)	0.0010 (5)
C13	0.0179 (7)	0.0180 (6)	0.0191 (7)	0.0022 (5)	0.0008 (5)	0.0045 (5)
C14	0.0166 (7)	0.0177 (6)	0.0242 (7)	0.0057 (5)	0.0048 (5)	0.0042 (5)
C15	0.0193 (7)	0.0118 (6)	0.0189 (6)	0.0024 (5)	0.0060 (5)	0.0018 (5)
C16	0.0305 (8)	0.0225 (7)	0.0227 (7)	0.0070 (6)	0.0018 (6)	0.0069 (6)
C17	0.0231 (7)	0.0251 (7)	0.0281 (7)	0.0102 (6)	0.0105 (6)	0.0011 (6)
C18	0.0335 (9)	0.0357 (9)	0.0194 (7)	0.0127 (7)	−0.0045 (6)	−0.0014 (6)
C19	0.0243 (7)	0.0323 (8)	0.0191 (7)	0.0121 (6)	0.0063 (6)	−0.0010 (6)

### Geometric parameters (Å, °)

**Table:**

O1—C1	1.2234 (16)	C6—H6A	0.9300
O2—N1	1.2257 (16)	C7—C8	1.3847 (19)
O3—N1	1.2257 (16)	C8—C9	1.3657 (19)
O4—C8	1.3651 (16)	C9—H9A	0.9300
O4—C16	1.4372 (17)	C10—C11	1.3946 (18)
O5—C7	1.3644 (16)	C10—C15	1.4009 (18)
O5—C16	1.4310 (17)	C11—C12	1.3941 (19)
O6—C15	1.3615 (15)	C12—C13	1.3874 (19)
O6—C17	1.4299 (16)	C12—H12A	0.9300
O7—C13	1.3649 (16)	C13—C14	1.3905 (19)
O7—C18	1.4280 (17)	C14—C15	1.3862 (19)
O8—C11	1.3677 (15)	C14—H14A	0.9300
O8—C19	1.4339 (16)	C16—H16A	0.9700
N1—C5	1.4637 (17)	C16—H16B	0.9700
C1—C2	1.4732 (18)	C17—H17A	0.9600
C1—C10	1.5007 (18)	C17—H17B	0.9600
C2—C3	1.3337 (19)	C17—H17C	0.9600
C2—H2A	0.9300	C18—H18A	0.9600
C3—C4	1.4680 (18)	C18—H18B	0.9600
C3—H3A	0.9300	C18—H18C	0.9600
C4—C5	1.4017 (19)	C19—H19A	0.9600
C4—C9	1.4092 (18)	C19—H19B	0.9600
C5—C6	1.3954 (19)	C19—H19C	0.9600
C6—C7	1.3642 (19)		
			
C8—O4—C16	106.10 (10)	O8—C11—C10	115.53 (11)
C7—O5—C16	106.10 (10)	C12—C11—C10	121.51 (12)
C15—O6—C17	117.41 (10)	C13—C12—C11	118.22 (12)
C13—O7—C18	117.45 (11)	C13—C12—H12A	120.9
C11—O8—C19	116.76 (10)	C11—C12—H12A	120.9
O2—N1—O3	123.06 (12)	O7—C13—C12	123.09 (12)
O2—N1—C5	117.74 (11)	O7—C13—C14	114.85 (12)
O3—N1—C5	119.18 (11)	C12—C13—C14	122.06 (12)
O1—C1—C2	119.91 (12)	C15—C14—C13	118.46 (12)
O1—C1—C10	120.19 (11)	C15—C14—H14A	120.8
C2—C1—C10	119.86 (11)	C13—C14—H14A	120.8
C3—C2—C1	123.32 (12)	O6—C15—C14	124.12 (12)
C3—C2—H2A	118.3	O6—C15—C10	114.43 (11)
C1—C2—H2A	118.3	C14—C15—C10	121.42 (12)
C2—C3—C4	124.64 (12)	O5—C16—O4	107.78 (10)
C2—C3—H3A	117.7	O5—C16—H16A	110.1
C4—C3—H3A	117.7	O4—C16—H16A	110.1
C5—C4—C9	117.26 (12)	O5—C16—H16B	110.1
C5—C4—C3	123.70 (12)	O4—C16—H16B	110.1
C9—C4—C3	118.94 (12)	H16A—C16—H16B	108.5
C6—C5—C4	124.53 (12)	O6—C17—H17A	109.5
C6—C5—N1	115.44 (11)	O6—C17—H17B	109.5
C4—C5—N1	120.02 (12)	H17A—C17—H17B	109.5
C7—C6—C5	115.53 (12)	O6—C17—H17C	109.5
C7—C6—H6A	122.2	H17A—C17—H17C	109.5
C5—C6—H6A	122.2	H17B—C17—H17C	109.5
C6—C7—O5	127.98 (12)	O7—C18—H18A	109.5
C6—C7—C8	121.86 (12)	O7—C18—H18B	109.5
O5—C7—C8	110.16 (12)	H18A—C18—H18B	109.5
O4—C8—C9	127.62 (12)	O7—C18—H18C	109.5
O4—C8—C7	109.79 (12)	H18A—C18—H18C	109.5
C9—C8—C7	122.58 (12)	H18B—C18—H18C	109.5
C8—C9—C4	118.24 (12)	O8—C19—H19A	109.5
C8—C9—H9A	120.9	O8—C19—H19B	109.5
C4—C9—H9A	120.9	H19A—C19—H19B	109.5
C11—C10—C15	118.31 (12)	O8—C19—H19C	109.5
C11—C10—C1	122.38 (11)	H19A—C19—H19C	109.5
C15—C10—C1	119.26 (11)	H19B—C19—H19C	109.5
O8—C11—C12	122.95 (12)		
			
O1—C1—C2—C3	−171.07 (12)	O1—C1—C10—C11	−114.47 (14)
C10—C1—C2—C3	6.82 (19)	C2—C1—C10—C11	67.64 (16)
C1—C2—C3—C4	175.54 (12)	O1—C1—C10—C15	62.88 (17)
C2—C3—C4—C5	153.75 (13)	C2—C1—C10—C15	−115.00 (13)
C2—C3—C4—C9	−29.9 (2)	C19—O8—C11—C12	4.76 (18)
C9—C4—C5—C6	−0.4 (2)	C19—O8—C11—C10	−176.43 (11)
C3—C4—C5—C6	176.01 (12)	C15—C10—C11—O8	−179.48 (11)
C9—C4—C5—N1	178.47 (12)	C1—C10—C11—O8	−2.09 (18)
C3—C4—C5—N1	−5.2 (2)	C15—C10—C11—C12	−0.65 (19)
O2—N1—C5—C6	−31.32 (18)	C1—C10—C11—C12	176.73 (12)
O3—N1—C5—C6	146.93 (13)	O8—C11—C12—C13	177.98 (11)
O2—N1—C5—C4	149.75 (14)	C10—C11—C12—C13	−0.76 (19)
O3—N1—C5—C4	−32.00 (19)	C18—O7—C13—C12	−11.05 (19)
C4—C5—C6—C7	0.4 (2)	C18—O7—C13—C14	168.92 (12)
N1—C5—C6—C7	−178.43 (11)	C11—C12—C13—O7	−178.33 (12)
C5—C6—C7—O5	179.77 (13)	C11—C12—C13—C14	1.7 (2)
C5—C6—C7—C8	−0.2 (2)	O7—C13—C14—C15	178.85 (11)
C16—O5—C7—C6	−179.08 (14)	C12—C13—C14—C15	−1.19 (19)
C16—O5—C7—C8	0.93 (15)	C17—O6—C15—C14	−11.24 (17)
C16—O4—C8—C9	178.84 (14)	C17—O6—C15—C10	166.67 (11)
C16—O4—C8—C7	−1.85 (16)	C13—C14—C15—O6	177.46 (11)
C6—C7—C8—O4	−179.39 (13)	C13—C14—C15—C10	−0.30 (19)
O5—C7—C8—O4	0.60 (17)	C11—C10—C15—O6	−176.77 (10)
C6—C7—C8—C9	0.0 (2)	C1—C10—C15—O6	5.76 (17)
O5—C7—C8—C9	179.95 (13)	C11—C10—C15—C14	1.19 (19)
O4—C8—C9—C4	179.36 (13)	C1—C10—C15—C14	−176.27 (11)
C7—C8—C9—C4	0.1 (2)	C7—O5—C16—O4	−2.04 (15)
C5—C4—C9—C8	0.05 (19)	C8—O4—C16—O5	2.40 (16)
C3—C4—C9—C8	−176.50 (12)		

### Hydrogen-bond geometry (Å, °)

**Table:**

*D*—H···*A*	*D*—H	H···*A*	*D*···*A*	*D*—H···*A*
C2—H2A···O1^i^	0.93	2.55	3.4512 (16)	164
C6—H6A···O6^ii^	0.93	2.36	3.2429 (16)	158

Symmetry codes: (i) −*x*, −*y*+1, −*z*; (ii) *x*, *y*+1, *z*.
